# Supplementary figures and images for: Identification of the best housekeeping gene for RT-qPCR analysis of human pancreatic organoids
Source: PLoS One. 2021 Dec 8;16(12):e0260902. doi: 10.1371/journal.pone.0260902 (PMC8654213; doi:10.1371/journal.pone.0260902)

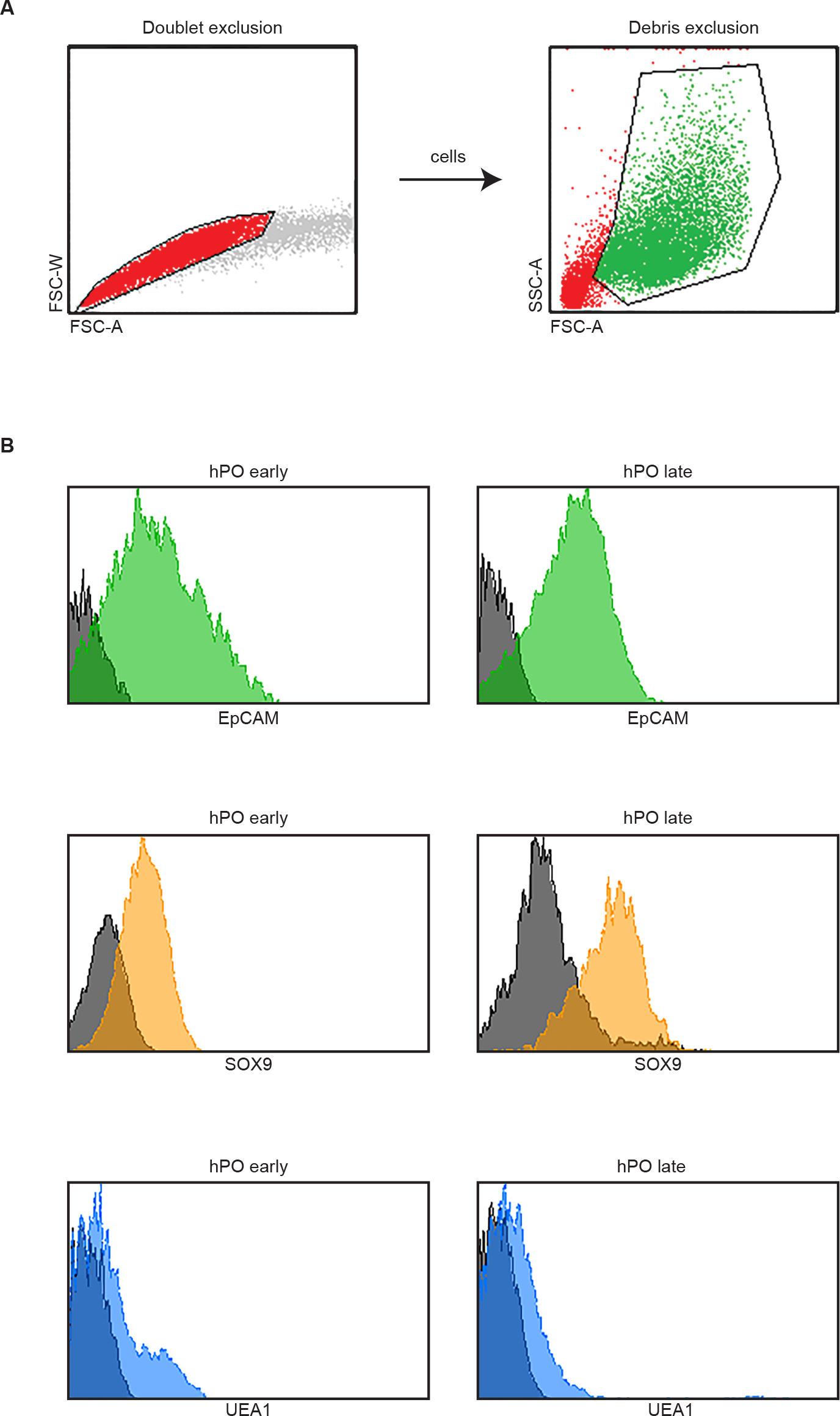

Supplement: S1 Fig — (TIF) [file pone.0260902.s001.tif]

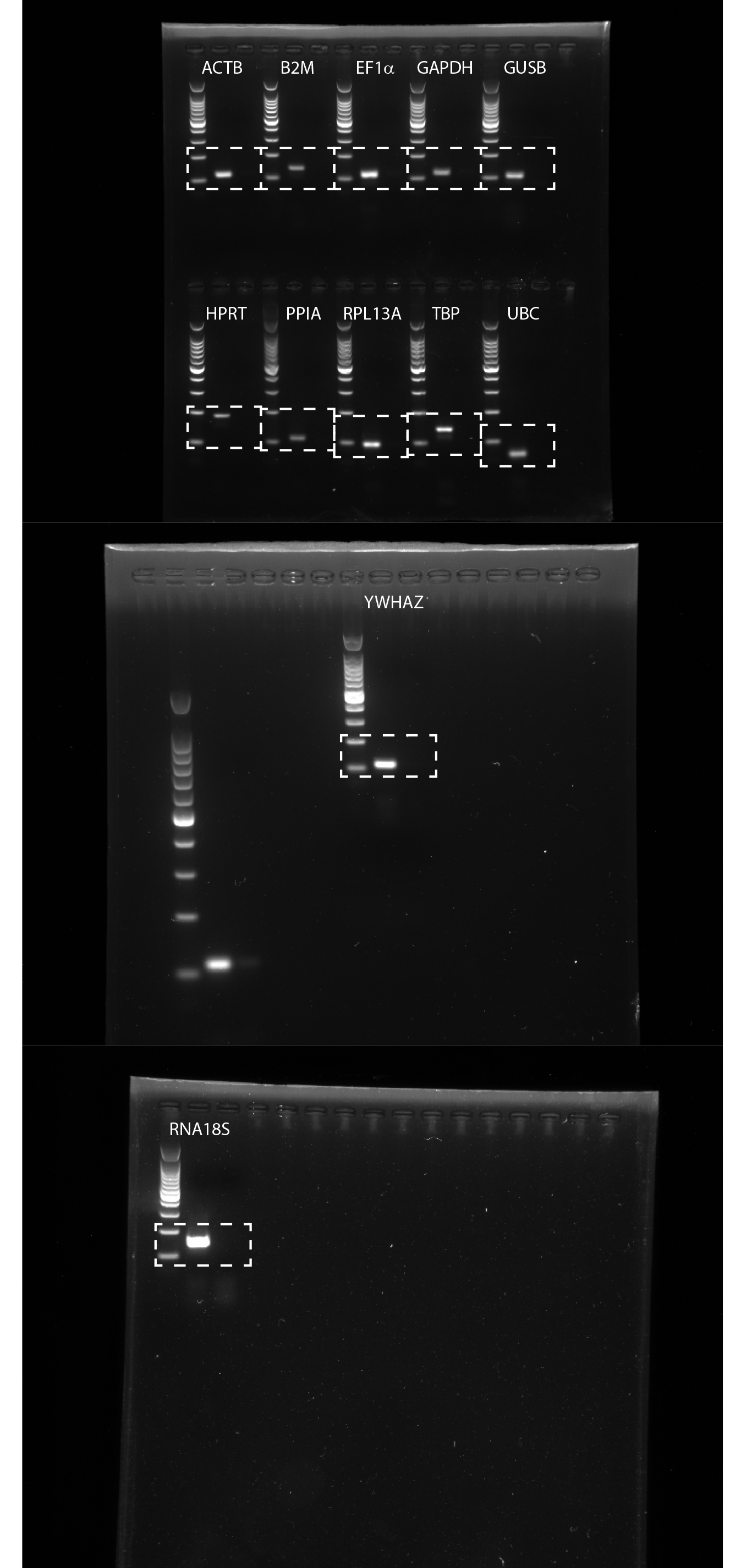

Supplement: S1 File — (ZIP) [file pone.0260902.s005.zip › Supporting information file_Cherubini et al/Uncropped gels/Uncropped gels relative to Figure 2.tif]
