# Supplementary material for: Identification of the best housekeeping gene for RT-qPCR analysis of human pancreatic organoids
Source: PLoS One. 2021 Dec 8;16(12):e0260902. doi: 10.1371/journal.pone.0260902 (PMC8654213; doi:10.1371/journal.pone.0260902)
Supplement: S1 Table — (DOCX) [file pone.0260902.s002.docx]

| **Gene** | **Full name** | **Accession number** | **Gene Cards** | **Function** | **Process** |
| --- | --- | --- | --- | --- | --- |
| ACTB | Actin beta | NM_001101.5 | https://www.genecards.org/cgi-bin/carddisp.pl?gene=ACTB | Cytoskeletal protein | Cell structure |
| B2M | Beta-2-Microglobulin | NM_004048.3 | https://www.genecards.org/cgi-bin/carddisp.pl?gene=B2M | Component of the class I major histocompatibility complex (MHC) | MHC-mediated immunity |
| EF1A | Eukaryotic Translation Elongation Factor 1 Alpha | NM_001402.6 | https://www.genecards.org/cgi-bin/carddisp.pl?gene=EEF1A1 | Responsible for the enzymatic delivery of aminoacyl tRNAs to the ribosome | Transcription |
| GAPDH | Glyceraldehyde-3-Phosphate Dehydrogenase | NM_002046.7 | https://www.genecards.org/cgi-bin/carddisp.pl?gene=GAPDH | Plays a role in glycolysis, gluconeogenesis and nuclear function | Glycolysis |
| GUSB | Glucuronidase Beta | NM_000181.4 | https://www.genecards.org/cgi-bin/carddisp.pl?gene=GUSB | Glycoprotein, exoglycosidase in lysosomes | Glycosylation |
| HPRT1 | Hypoxanthine Phosphoribosyltransferase 1 | NM_000194.3 | https://www.genecards.org/cgi-bin/carddisp.pl?gene=HPRT1 | Converts guanine to guanosine monophosphate and hypoxanthine to inosine monophosphate | Nucleotide-metabolism |
| PPIA | Peptidylprolyl Isomerase A | NM_021130.5 | https://www.genecards.org/cgi-bin/carddisp.pl?gene=PPIA | Catalyzes the cis-trans isomerization of proline imidic peptide bonds. | Protein folding |
| RNA18S | 18 S Ribosomal RNA | NR_003286.4 | https://www.genecards.org/cgi-bin/carddisp.pl?gene=RNA18SN5 | Component of the small eukaryotic ribosomal subunit (40S). | Translation |
| RPL13A | Ribosomal Protein L13a | NM_012423.4 | https://www.genecards.org/cgi-bin/carddisp.pl?gene=RPL13A | Part of a ribosomal subunit | Translation |
| TBP | TATA-Box Binding Protein | NM_003194.5 | https://www.genecards.org/cgi-bin/carddisp.pl?gene=TBP | Component of the core of the transcription factor IID (TFIID) | Transcription |
| UBC | Ubiquitin C | NM_021009.7 | https://www.genecards.org/cgi-bin/carddisp.pl?gene=UBC | Maintains cellular ubiquitin levels under stress conditions | Proteolysis |
| YWHAZ | 14-3-3 Protein Zeta/Delta | NM_003406.4 | https://www.genecards.org/cgi-bin/carddisp.pl?gene=YWHAZ | Regulates cell survival and interacts with many apoptotic proteins | Metabolism, apoptosis and cell cycle regulation |

**Supplementary Table 1.** Candidate HKGs
