# Supplementary material for: Identification of the best housekeeping gene for RT-qPCR analysis of human pancreatic organoids
Source: PLoS One. 2021 Dec 8;16(12):e0260902. doi: 10.1371/journal.pone.0260902 (PMC8654213; doi:10.1371/journal.pone.0260902)
Supplement: S2 Table — (DOCX) [file pone.0260902.s003.docx]

| GENE | PRIMER | SEQUENCE | PRODUCT LENGTH | EFFICIENCY | SLOPE |
| --- | --- | --- | --- | --- | --- |
| ACTB | Forward | CACGATGGAGGGGAAGACGG | 120 bp | 97.5% | -3.383 |
|  | Reverse | CGCCGCCAGCTCACCATG |  |  |  |
| B2M | Forward | CATTCCTGAAGCTGACAGCATTC | 136 bp | 95.0% | -3.448 |
|  | Reverse | TGCTGGATGACGTGAGTAAACC |  |  |  |
| EF1α | Forward | CCGCCAGAACACAGGTGTCGT | 118 bp | 97.4% | -3.387 |
|  | Reverse | CCAGTAGTGGTGGACTTGCCCGA |  |  |  |
| GAPDH | Forward | AGGTGAAGGTCGGAGTCAAC | 123 bp | 98.6% | -3.355 |
|  | Reverse | CCATGTAGTTGAGGTCAATGAAG |  |  |  |
| GUSB | Forward | AGTGCAAGGAGCTGGACGGC | 112 bp | 99.9% | -3.324 |
|  | Reverse | GTGGGGCCTGACTCCCACA |  |  |  |
| HPRT1 | Forward | CTGGCGTCGTGATTAGTG | 185 bp | 96.1% | -3.420 |
|  | Reverse | CACACAGAGGGCTACAATG |  |  |  |
| PPIA | Forward | ACGCCACCGCCGAGGAAAAC | 113 bp | 97.0% | -3.396 |
|  | Reverse | TGCAAACAGCTCAAAGGAGACGC |  |  |  |
| 18S | Forward | AACCCGTTGAACCCCATT | 149 bp | 104.4% | -3.220 |
|  | Reverse | CCATCCAATCGGTAGTAGCG |  |  |  |
| RPL13A | Forward | GGCTTTCCTCCGCAAGCGGAT | 101 bp | 96.8% | -3.400 |
|  | Reverse | GCAGCATACCTCGCACGGTCC |  |  |  |
| TBP | Forward | GCCACGCCAGCTTCGGAGAG | 145 bp | 102.6% | -3.262 |
|  | Reverse | CCGCAGCAAACCGCTTGGGA |  |  |  |
| UBC | Forward | TAGTTCCGTCGCAGCCGGGA | 72 bp | 98.5% | -3.357 |
|  | Reverse | GCATTGTCAAGTGACGATCACAGCG |  |  |  |
| YWHAZ | Forward | CCGCTGGTGATGACAAGAAAGGGAT | 118 bp | 100.1% | -3.319 |
|  | Reverse | AGGGCCAGACCCAGTCTGATAGGA |  |  |  |

**Supplementary Table 2.** Primer sequences, amplicon length and primer efficiencies
