# Supplementary material for: Identification of the best housekeeping gene for RT-qPCR analysis of human pancreatic organoids
Source: PLoS One. 2021 Dec 8;16(12):e0260902. doi: 10.1371/journal.pone.0260902 (PMC8654213; doi:10.1371/journal.pone.0260902)
Supplement: S3 Table — (DOCX) [file pone.0260902.s004.docx]

| **Gene** | **hPO P0 1** | **hPO P0 2** | **hPO P0 3** | **hPO P2 1** | **hPO P2 2** | **hPO P2 3** | **hPO P5 1** | **hPO P5 2** | **hPO P5 3** | **hPO P7 1** | **hPO P7 2** | **hPO P7 3** | **hPO P10 1** | **Hpo P10 2** | **Hpo P10 3** | **Average** | **Standard deviation** | **%CV** |
| --- | --- | --- | --- | --- | --- | --- | --- | --- | --- | --- | --- | --- | --- | --- | --- | --- | --- | --- |
| **RNA18S** | 11,27 | 10,25 | 11,11 | 10,76 | 8,19 | 8,60 | 9,76 | 9,73 | 9,71 | 10,32 | 8,71 | 8,22 | 9,02 | 10,36 | 9,94 | 9,73 | 1,00 | 10,23 |
| **ACTB** | 23,31 | 22,93 | 22,51 | 23,30 | 22,76 | 23,27 | 22,01 | 20,63 | 21,18 | 21,77 | 22,07 | 20,10 | 20,31 | 21,91 | 20,18 | 21,88 | 1,16 | 5,31 |
| **B2M** | 20,08 | 20,51 | 19,23 | 21,17 | 22,31 | 21,22 | 22,43 | 21,14 | 21,12 | 19,79 | 21,71 | 22,37 | 20,35 | 22,18 | 20,10 | 21,05 | 1,02 | 4,84 |
| **EF1a** | 18,87 | 19,49 | 18,28 | 18,03 | 20,00 | 18,60 | 20,40 | 19,63 | 20,00 | 17,76 | 19,67 | 20,15 | 19,04 | 21,12 | 18,93 | 19,33 | 0,94 | 4,87 |
| **GAPDH** | 19,42 | 20,39 | 20,51 | 19,16 | 19,84 | 18,59 | 19,55 | 18,59 | 18,67 | 18,57 | 19,76 | 19,38 | 18,59 | 20,59 | 18,31 | 19,33 | 0,77 | 3,99 |
| **GUSB** | 27,64 | 27,59 | 27,05 | 27,62 | 29,21 | 28,48 | 28,82 | 27,19 | 27,52 | 27,42 | 29,03 | 28,38 | 26,30 | 29,41 | 26,75 | 27,89 | 0,94 | 3,38 |
| **HPRT** | 25,87 | 26,24 | 25,58 | 24,72 | 26,64 | 26,16 | 26,67 | 25,56 | 26,23 | 24,40 | 26,32 | 26,48 | 24,71 | 26,69 | 24,79 | 25,80 | 0,80 | 3,09 |
| **PPIA** | 22,60 | 23,01 | 22,95 | 22,05 | 23,41 | 22,64 | 23,17 | 20,98 | 21,83 | 22,29 | 23,47 | 22,50 | 21,04 | 22,47 | 20,86 | 22,35 | 0,85 | 3,82 |
| **RPL13A** | 21,02 | 21,77 | 21,53 | 20,53 | 22,43 | 21,23 | 22,67 | 21,24 | 21,43 | 21,00 | 22,06 | 21,98 | 20,09 | 22,20 | 20,28 | 21,43 | 0,77 | 3,60 |
| **TBP** | 31,46 | 32,00 | 31,01 | 31,60 | 32,75 | 33,11 | 32,78 | 30,83 | 31,97 | 30,59 | 32,37 | 32,67 | 29,29 | 32,66 | 30,28 | 31,69 | 1,10 | 3,49 |
| **UBC** | 24,25 | 23,96 | 23,06 | 24,33 | 24,32 | 23,30 | 26,09 | 23,76 | 25,08 | 22,41 | 23,80 | 24,14 | 23,49 | 25,58 | 24,02 | 24,11 | 0,94 | 3,91 |
| **YWHAZ** | 31,54 | 32,14 | 30,86 | 31,63 | 33,83 | 32,81 | 33,94 | 32,02 | 31,56 | 29,56 | 31,91 | 32,92 | 30,24 | 32,58 | 30,70 | 31,88 | 1,24 | 3,87 |

**Supplementary table 3.** Coefficient of variation of selected HKGs.
