# Supplementary material for: Identification of the best housekeeping gene for RT-qPCR analysis of human pancreatic organoids
Source: PLoS One. 2021 Dec 8;16(12):e0260902. doi: 10.1371/journal.pone.0260902 (PMC8654213; doi:10.1371/journal.pone.0260902)
Supplement: Attachment — Submitted filename: Rebuttal Letter_Cherubini et al.doc [file pone.0260902.s006.doc]

**Journal Requirements:**

When submitting your revision, we need you to address these additional requirements.

1. Please ensure that your manuscript meets PLOS ONE's style requirements, including those for file naming. The PLOS ONE style templates can be found at

https://journals.plos.org/plosone/s/file?id=wjVg/PLOSOne_formatting_sample_main_body.pdf

https://journals.plos.org/plosone/s/file?id=ba62/PLOSOne_formatting_sample_title_authors_affiliations.pdf

We formatted our manuscript following the PLOS ONE's style requirements.

2. Please amend your current ethics statement to address the following concerns:

a) Did participants provide their written or verbal informed consent to participate in this study?

b) If consent was verbal, please explain i) why written consent was not obtained, ii) how you documented participant consent, and iii) whether the ethics committees/IRB approved this consent procedure.

Human pancreata were obtained from the Pancreatic Islet Processing Unit of the Diabetes Research Institute, IRCCS Ospedale San Raffaele, Milan, Italy, in the context of transplantation organ, after approval of the Institutional Review Board (National Transplant Center accredited facility IT000679). Therefore, the fresh tissue was obtained from organ donors. The use of human specimens was approved by the Ethical Committee of Fondazione IRCCS Ca’ Granda Ospedale Maggiore Policlinico n° 1982, 14th January 2020.

3. Thank you for stating the following financial disclosure:

[This work was funded by the grant “LSFM4LIFE–Production and characterization of endocrine cells derived from human pancreas organoids for the cell-based therapy of Type 1 diabetes”, project number 668350.]

Please state what role the funders took in the study. If the funders had no role, please state: "The funders had no role in study design, data collection and analysis, decision to publish, or preparation of the manuscript."

If this statement is not correct you must amend it as needed.

Please respond by return e-mail so that we can amend your financial disclosure and competing interests on your behalf.

The funders had no role in study design, data collection and analysis, decision to publish, or preparation of the manuscript. Therefore, we added the appropriate sentences as suggested.

4. Thank you for stating the following in the Acknowledgments Section of your manuscript:

[hPOs were generated starting from adult healthy islet-depleted pancreatic tissue gently provided by the Diabetes Research Institute, IRCCS Ospedale San Raffaele, Milan, Italy. This work was funded by the grant “LSFM4LIFE–Production and characterization of endocrine cells derived from human pancreas organoids for the cell-based therapy of Type 1 diabetes”, project number 668350.]

We note that you have provided funding information that is not currently declared in your Funding Statement. However, funding information should not appear in the Acknowledgments section or other areas of your manuscript. We will only publish funding information present in the Funding Statement section of the online submission form.

Please remove any funding-related text from the manuscript and let us know how you would like to update your Funding Statement. Currently, your Funding Statement reads as follows:

[This work was funded by the grant “LSFM4LIFE–Production and characterization of endocrine cells derived from human pancreas organoids for the cell-based therapy of Type 1 diabetes”, project number 668350.]

Please include your amended statements within your cover letter; we will change the online submission form on your behalf.

We included the acknowledgment section in cover letter as suggested.

5. PLOS ONE now requires that authors provide the original uncropped and unadjusted images underlying all blot or gel results reported in a submission’s figures or Supporting Information files. This policy and the journal’s other requirements for blot/gel reporting and figure preparation are described in detail at https://journals.plos.org/plosone/s/figures#loc-blot-and-gel-reporting-requirements and https://journals.plos.org/plosone/s/figures#loc-preparing-figures-from-image-files. When you submit your revised manuscript, please ensure that your figures adhere fully to these guidelines and provide the original underlying images for all blot or gel data reported in your submission. See the following link for instructions on providing the original image data: https://journals.plos.org/plosone/s/figures#loc-original-images-for-blots-and-gels.

In your cover letter, please note whether your blot/gel image data are in Supporting Information or posted at a public data repository, provide the repository URL if relevant, and provide specific details as to which raw blot/gel images, if any, are not available. Email us at plosone@plos.org if you have any questions.

We generated a supporting information that contains all uncropped gels underline the picture used in our manuscript and related to Figure 2.

6. PLOS requires an ORCID iD for the corresponding author in Editorial Manager on papers submitted after December 6th, 2016. Please ensure that you have an ORCID iD and that it is validated in Editorial Manager. To do this, go to ‘Update my Information’ (in the upper left-hand corner of the main menu), and click on the Fetch/Validate link next to the ORCID field. This will take you to the ORCID site and allow you to create a new iD or authenticate a pre-existing iD in Editorial Manager. Please see the following video for instructions on linking an ORCID iD to your Editorial Manager account: <https://www.youtube.com/watch?v=_xcclfuvtxQ>

We added the ORCID ID of all the authors into the cover letter.

7. We note that you have included the phrase “data not shown” in your manuscript. Unfortunately, this does not meet our data sharing requirements. PLOS does not permit references to inaccessible data. We require that authors provide all relevant data within the paper, Supporting Information files, or in an acceptable, public repository. Please add a citation to support this phrase or upload the data that corresponds with these findings to a stable repository (such as Figshare or Dryad) and provide and URLs, DOIs, or accession numbers that may be used to access these data. Or, if the data are not a core part of the research being presented in your study, we ask that you remove the phrase that refers to these data.

We removed the sentence “data not shown” and we generated a supporting information containing all the sequences used in our manuscript.

8. Please include captions for your Supporting Information files at the end of your manuscript, and update any in-text citations to match accordingly. Please see our Supporting Information guidelines for more information: <http://journals.plos.org/plosone/s/supporting-information>.

We added captions related to our supporting information at the end of the paper after Reference section as reported here:

Supporting information

Fig. S1 Flow cytometry analysis of human pancreatic organoids.

Table S1 Candidate HKGs.

Table S2 Primer sequences, amplicon length and primer efficiencies.

Table S3 Coefficient of variation of selected HKGs.

Supporting information file Sanger sequencing and uncropped gel images.

**Reviewer #1:** In this report, Alessandro Cherubini and colleagues present data on gene expression changes occurring during pancreas related organoid culture maturation. These results are potentially quite interesting, and it has strong scientific value which can be applied to identify the most reliable control for gene expression studies. However, they well-performed experiments to try to answer these specific questions, my feeling is that two time point limiting the evaluation of their result so I recommend additional time points to extend their study. My feeling is that although it is an interesting study with a valuable scientific background, the presented results must be supplemented with additional measurements:

The authors use an early and a late time point to measure gene expression of several housekeeping gene. I would strongly recommend including to the study the original primary cell which they used to create the organoid culture. Additionally, I also strongly suggest adding 2-3 time points between the early and late stage to prove that the development of the organoid does not affect significantly the expression of the selected genes.

We thank Reviewer#1 for these comments. Following his/her indication we added the results obtained using the original primary cells from which we generated our hPOs (named P0 in our revised manuscript) after removing the islet-depleted tissue. The pancreatic tissue mainly consists of acinar cells, whereas organoids are almost exclusively composed by ductal cells and this could be considered misleading. Furthermore, to reinforce our analysis we added other time points (P0, P2, P7), and now the gap between two time points is no longer than three passages, we made it to avoid the effect of the development of hPOs on the expression of our selected genes.

Finally, I also strongly recommend changing the title form a general one to more specific-one

We are grateful for this recommendation. The new title that we proposed is: “Identification of the best housekeeping gene for RT-qPCR analysis of human pancreatic organoids.”.

Based on these I recommend major revision of the current study before publication.

**Reviewer #2:** Cherubini et al. analysed the expression of housekeeping genes in human pancreatic organoids. This is an important contribution as 3D organoid culture is an emerging technology that is utilized in a rapidly increasing number of studies. The paper is well executed and the results are analysed correctly.

**Major comment:**

1. The authors used the islet-depleted pancreatic tissue to compare changes of HKGs in early and late organoids. This is somewhat misleading, as the pancreatic tissue consist mostly of acinar cells, whereas organoids are almost exclusively ductal cells. Therefore comparing isolated ductal fragments with organoids seems to be more relevant.

We thank Reviewer#2 for these comments. Following his/her advice we decided to remove the islet-depleted pancreatic tissue from our analysis, and we replaced it with the hPO passage 0 (P0) because it is enriched of ductal fragments from which organoids are derived as suggested in our previously published paper: “Standardized GMP-compliant scalable production of human pancreas organoids”, Dossena et al., 2020 DOI: 10.1186/s13287-020-1585-2.

2. How was "early' and "late" passages defined? The early passage in my point of view would be passage No. 2. In the fifth passage the organoids were grown in vitro for several weeks, which may impact gene expression. Therefore I recommend adding passage No.2. to the anaylsis as well.

We are grateful for these comments that improved our manuscript. Since human pancreatic organoids could be expanded for several months, we considered at “early passage” hPO at P5 (< 2 months), and at “late passage” the hPO cultured for more than P5 (> 2 months). We reported the description of early and late passages in the caption of Figure 1. We totally agree with Reviewer#2 that hPO at P2 are the best representation of an “early passage”, therefore we included in our study also the hPO at P2. Moreover, to exclude that the development of the organoid did affect significantly the expression of our selected genes we added other passages. In the present form, different time points (P0, P2, P5, P7, P10) are presented, therefore now the gap between the two time points is no longer than three passages.

**Minor point:**

1. The "early" and "late" organoids are described in section two in the Results, however it is already used in Figure 1.

We added the definition of “early” and “late” passage in the main text, Figure 1 caption (lines 159-163 revised manuscript and lines 154-158 manuscript).
